# Epithelioid Sarcoma: A Review and Update

**DOI:** 10.3390/cancers18162717

**Published:** 2026-08-21

**Authors:** Jun Nishio, Shizuhide Nakayama, Mikiko Aoki

**Affiliations:** 1 Section of Orthopaedic Surgery, Department of Medicine, Fukuoka Dental College, 2-15-1 Tamura, Sawara-ku, Fukuoka 814-0193, Japan; 2 Nakayama Orthopaedic Clinic, 6-10-3 Umebayashi, Sawara-ku, Fukuoka 814-0172, Japan; n.shizuhide@gmail.com; 3 Department of Pathology, Faculty of Medicine, Fukuoka University, 7-45-1 Nanakuma, Jonan-ku, Fukuoka 814-0180, Japan; mikikoss@fukuoka-u.ac.jp

**Keywords:** epithelioid sarcoma, SMARCB1, INI1, diagnosis, pathogenesis, treatment

## Abstract

Epithelioid sarcoma (EPS) is an ultra-rare malignant mesenchymal neoplasm of uncertain differentiation that comprises two distinct clinicopathological subtypes: classic and proximal. Classic EPS most commonly occurs in the distal upper extremity of adolescents and young adults, whereas proximal-type EPS most often affects the truncal regions of young to middle-aged adults. Both classic and proximal-type EPSs exhibit aggressive clinical behavior, including a higher risk of local recurrence and regional lymph node or distant metastasis. Histologically, classic EPS is characterized by irregular nodules composed of epithelioid and spindled cells, while proximal-type EPS consists of multinodular distributions and sheets of large polygonal cells. EPS has a distinctive immunoprofile with characteristic expression of cytokeratins and epithelial membrane antigen. Loss of nuclear expression of SMARCB1 protein occurs in the vast majority of cases. Moreover, *SMARCB1* homozygous deletions have also been observed in both subtypes. Surgery is the mainstay treatment approach for localized EPS. Systemic treatment options for metastatic or unresectable locally advanced disease are very limited. The withdrawal of tazemetostat has created a significant gap in available treatment options. In this review, we provide an overview of the current knowledge on the clinical and radiological features, histopathology, immunohistochemistry, pathogenesis, and management of EPS.

## Simple Summary

Epithelioid sarcoma (EPS) represents a SMARCB1-deficient mesenchymal neoplasm displaying epithelioid cytomorphology and predominantly epithelial differentiation. Conventionally, EPS is histologically classified as either the classic or proximal subtype, each exhibiting distinct clinical behavior. Loss of SMARCB1 is an important diagnostic feature, but it is neither entirely specific nor an established predictive biomarker. There is no strong evidence of systemic therapy for advanced/metastatic EPS. Tazemetostat is no longer routinely available. Our objective with this review is to provide a comprehensive reference, offer valuable insights into the clinicopathological and molecular characteristics, and summarize current therapeutic options for EPS.

## 1. Introduction

Epithelioid sarcoma (EPS) is an extremely rare and aggressive mesenchymal neoplasm showing uncertain but multidirectional, predominantly epithelial differentiation. It was first described as a sarcoma simulating a granuloma or carcinoma by Enzinger in 1970 [1]. The overall incidence of EPS is 0.03–0.041 cases per 100,000 individuals [2,3]. The etiology of this neoplasm is uncertain, with a largely unknown natural history [3]. EPS can often be difficult to diagnose because of its rarity and morphological overlap with diverse mimics. A hallmark of EPS is the loss of switch/sucrose nonfermentable (SWI/SNF)-related BAF chromatin remodeling complex subunit B1 (SMARCB1) nuclear protein expression [4]. Surgery remains the mainstay of treatment for localized EPS. There is currently a lack of consensus regarding the optimal treatment strategy for metastatic or unresectable locally advanced disease.

A comprehensive narrative review of the literature indexed in PubMed, MEDLINE, and Scopus databases was performed from inception through June 2026. Search terms included “epithelioid sarcoma”, “SMARCB1”, “immunohistochemistry”, “cytogenetics”, “genetics”, “surgery”, “systemic therapy”, “chemotherapy”, “radiotherapy”, and “tazemetostat”, using various Boolean combinations. The reference lists of relevant studies were screened to identify other studies of interest. We excluded non-English publications and conference abstracts for which no full-text article was available.

In this review, we provide an updated overview of the clinical, pathological, and molecular landscape of EPS, focusing on the molecular distinction between classic and proximal-type EPSs, the emerging immune landscape, and the withdrawal of tazemetostat.

## 2. Clinical Features and Prognosis

EPS constitutes less than 1% of all adult soft tissue sarcomas (STSs) [2] and accounts for approximately 4–8% of pediatric nonrhabomyosarcomatous STSs [5]. There are two distinct clinicopathological EPS subtypes: the classic (or distal) type and the more recently identified proximal (or large cell) type [4]. Classic EPS most commonly occurs in the distal upper extremity, particularly the fingers and hand, of adolescents and young adults, with a male predominance [4]. It typically presents as a superficial, usually painless, slow-growing, firm nodule, often accompanied by ulceration or necrosis. Multiple nodules representing local disease spread may be present at the time of diagnosis. Proximal-type EPS most often affects the truncal regions and tends to occur in older adults (range, 13 to 80 years; mean, 40 years), with a slight male predominance [6,7]. A palpable mass (range, 2 to 16 cm; mean, 7.8 cm) is the main presenting symptom of proximal-type EPS [7]. Compared with classic EPS, proximal-type EPS tends to arise in the deep soft tissue and is somewhat larger in size. The mean duration from symptom onset to diagnosis is 7.1–36 months [8]. Interestingly, approximately 20–27% of cases are associated with antecedent trauma [1,9,10].

Cancer antigen 125 (CA125), also known as mucin 16 (MUC16), has been reported as a potential biomarker in both classic and proximal-type EPSs [11,12,13,14]. Elevated serum CA125 levels have been shown to correlate with disease progression [11]. Moreover, one study demonstrated strong CA125 protein expression in EPS tissues compared with that in other STSs using immunohistochemistry assays [15]. Most recently, Connolly et al. reported the efficacy of ubamatamab, a bispecific antibody targeting MUC16 and CD3, in a single patient with MUC16-positive metastatic EPS [14]. However, more evidence is required to determine whether CA125 can serve as a useful biomarker for diagnosis, surveillance, or treatment selection in EPS.

Notably, EPS has an aggressive clinical course with a high risk of local recurrence and metastatic spread. The prognosis of patients with proximal-type EPS is significantly poorer than that of patients with classic EPS [7,16]. Local recurrence occurs in 35–77% of cases [3,10,17,18], often repeatedly and necessitating amputation. Metastasis develops in 40–61% of EPS cases [3,10,17,18,19], with the lung being the most common distant metastatic site [18]. Unlike other STSs, EPS shows a high propensity for regional lymph node metastasis, ranging from 23% to 48% [2,10,18]. Large population-based studies using the Surveillance, Epidemiology, and End Results (SEER) database have indicated that the 5- and 10-year survival rates are 50–68% and 50–61%, respectively [2,17,19,20]. Pediatric patients generally have a more favorable prognosis, with a 5-year survival rate of 92.4% and a 10-year survival rate of 86.9% [5]. The possible prognostic factors include age at initial diagnosis, tumor size, tumor site, histological grade, the presence of rhabdoid cells, and the presence of lymph node and distant metastases [4,21].

## 3. Radiological Features

A variety of imaging modalities have been applied for EPS detection and follow-up. Radiographs may be normal or show a nonspecific soft tissue mass. The underlying bone is usually normal, with calcification observed in approximately 20–28% of cases [10,22]. Ultrasonography may reveal an ill-defined, lobulated hypoechoic mass with internal vascularity [23]. Computed tomography (CT) demonstrates a soft tissue mass that has an attenuation slightly less than or equal to that of skeletal muscle, with multilobulated contours [22,24]. On magnetic resonance imaging (MRI), lesions are usually isointense relative to skeletal muscle on T1-weighted sequences and heterogeneously hyperintense relative to skeletal muscle on T2-weighted sequences. Peritumoral edema may be seen [25,26]. Regional lymphadenopathy can sometimes be observed [25]. Contrast-enhanced MRI demonstrates intense heterogeneous or homogeneous enhancement [26]. Integrated positron emission tomography (PET)/CT shows increased fluorodeoxyglucose (FDG) uptake in the lesion [23,27,28,29,30]. The mean SUVmax (maximum standardized uptake value) for EPS is 6.9 [27].

## 4. Histological and Immunohistochemical Features

Grossly, EPSs usually appear as ill-defined, solitary or multiple nodules with gray-white, glistening cut surfaces. Apparent areas of hemorrhage and necrosis can often be seen in both classic and proximal-type EPSs [7,10].

**Figure 1:**
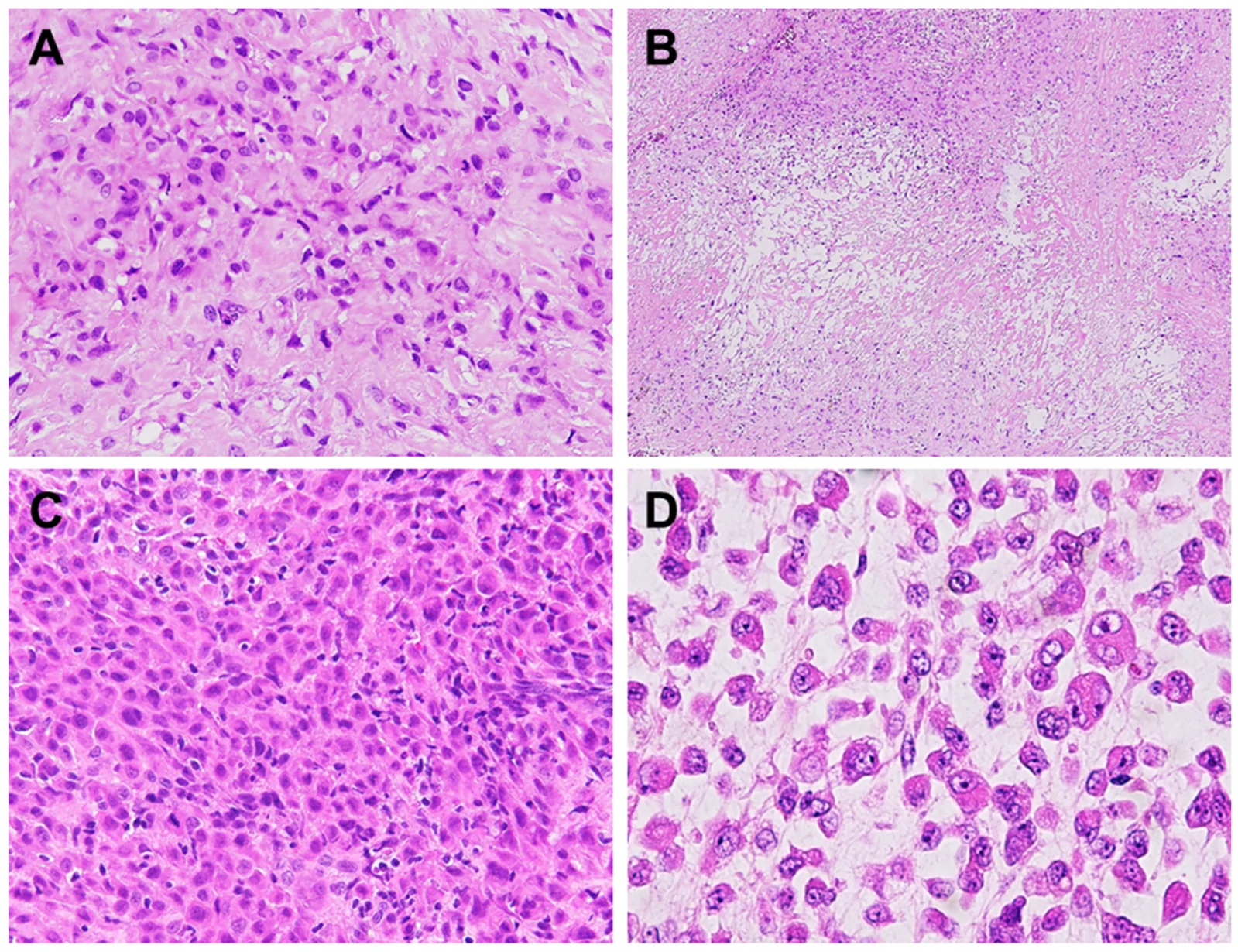
Histological findings of classic and proximal-type epithelioid sarcomas (EPSs) with the use of hematoxylin and eosin staining: (**A**) Classic EPS is composed of epithelioid and spindle-shaped cells with abundant eosinophilic cytoplasm. (**B**) Central necrosis can be seen. (**C**) Proximal-type EPS is composed of large polygonal cells with abundant eosinophilic cytoplasm and enlarged vesicular nuclei. (**D**) Rhabdoid cells can be seen.

Classic EPS is characterized by cellular nodules composed of a proliferation of epithelioid cells and plump spindle-shaped cells with a deeply eosinophilic cytoplasm and mildly atypical nuclei with vesicular chromatin and small nucleoli [4] (Figure 1A). Tumor nodules often show central necrosis (Figure 1B), mimicking necrobiotic granuloma. Dystrophic calcification and metaplastic bone formation are observed in 19% and 10% of cases, respectively [10]. Aggregates of chronic inflammatory cells are often observed. Mitotic activity is usually low. Proximal-type EPS is characterized by multinodular and sheet-like growth of large polygonal cells with enlarged vesicular nuclei and prominent nucleoli [4] (Figure 1C). Compared with classic EPS, proximal-type EPS more frequently displays rhabdoid morphology (Figure 1D). A pseudoangiosarcomatous pattern can occasionally be present [6]. The mitotic activity ranges from 2 to 20 per 10 high-power fields [6].

**Figure 2:**
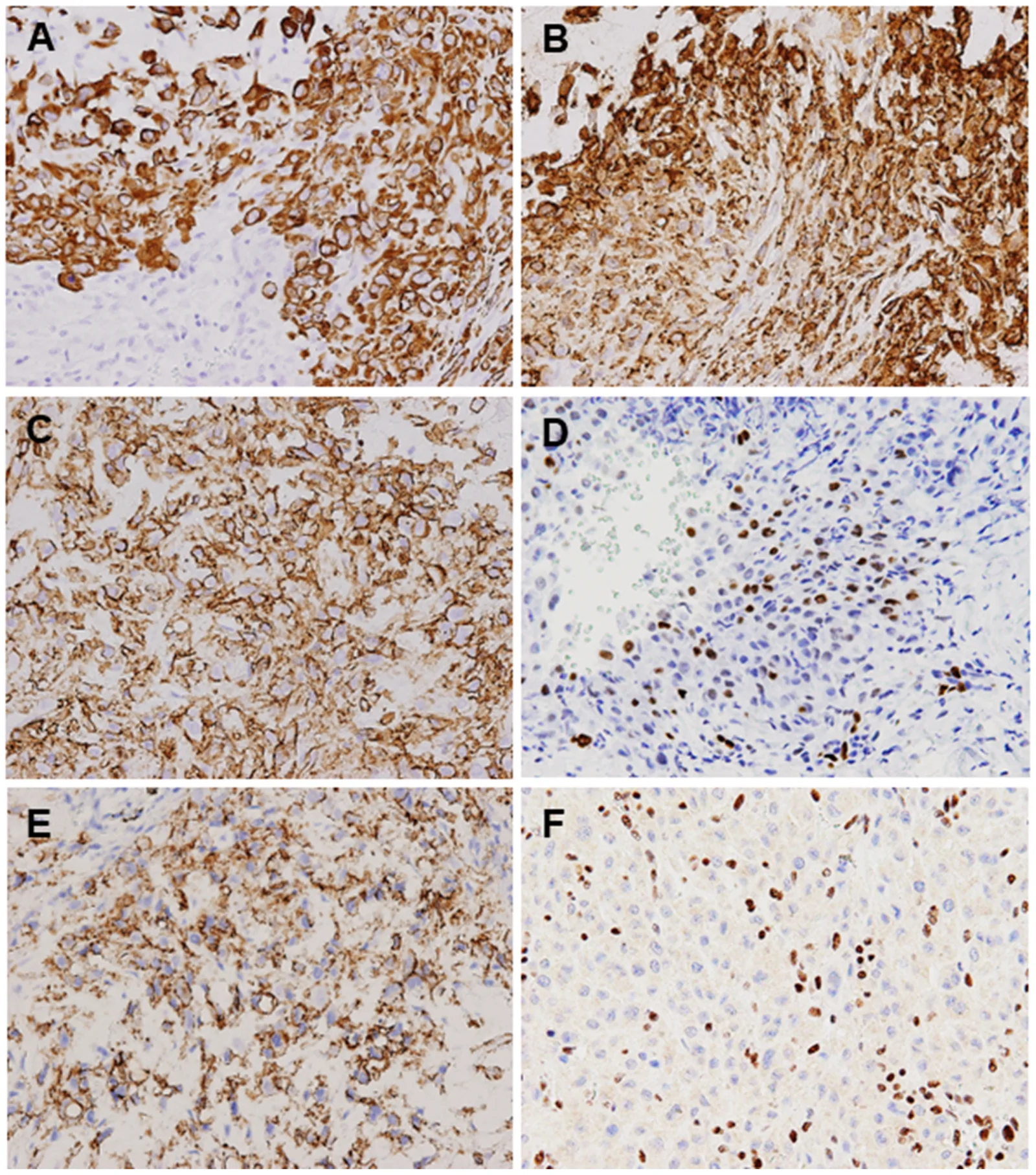
Immunohistochemistry of epithelioid sarcoma. The neoplastic cells are strongly positive for cytokeratin (AE1/AE3) (**A**) and EMA (**B**). In addition, the neoplastic cells are diffusely positive for CD34 (**C**) and focally positive for ERG (**D**) and CA125 (**E**). Moreover, loss of SMARCB1 nuclear expression is observed in the neoplastic cells, whereas infiltrating lymphocytes or vascular endothelial cells disclose immunoreactivity (**F**).

Immunohistochemically, the neoplastic cells show positive expression of epithelial markers, including low- and high-molecular-weight cytokeratins (Figure 2A) and epithelial membrane antigen (EMA) (Figure 2B). CD34 is expressed in 52–73% of cases [31,32,33] (Figure 2C). In addition, expression of ETS transcription factor ERG (ERG) (Figure 2D) and Fli-1 proto-oncogene, ETS transcription factor (FLI1) is observed in 38–68% and 93% of cases, respectively [33,34]. As mentioned above, CA125 is strongly expressed in approximately 91% of cases [15] (Figure 2E). Most notably, loss of SMARCB1 nuclear expression is seen in over 90% of cases of both types [35] (Figure 2F). However, loss of SMARCB1 nuclear expression is not entirely specific because it may also occur in histological mimics of EPS. The MIB-1 labeling index is variable, but significantly higher, in proximal-type EPS compared with that in classic EPS [36].

The histological differential diagnosis of EPS is notably broad and includes necrobiotic granuloma, undifferentiated carcinoma, extrarenal rhabdoid tumor (ERRT), epithelioid angiosarcoma, pseudomyogenic hemangioendothelioma (PHE), epithelioid malignant peripheral nerve sheath tumor (MPNST), synovial sarcoma, melanoma, and myoepithelial carcinoma. In our experience, necrobiotic granuloma can be confused with EPS. Unlike EPS, necrobiotic granuloma lacks expression of cytokeratins and EMA [37]. Undifferentiated carcinoma occasionally displays an abundant eosinophilic cytoplasm, closely resembling EPS. However, most carcinomas lack expression of CD34 [38]. ERRT is most frequently confused with EPS, particularly the proximal type. Compared with EPS, ERRT tends to occur in infants and young children, often expressing glypican 3 (GPC3) and spalt-like transcription factor 4 (SALL4) [39,40]. Moreover, ERRT typically lacks ERG expression, unlike EPS [40]. While epithelioid angiosarcoma may also have overlapping histological features with EPS, it expresses CD31 and shows retention of SMARCB1 expression [34,38]. Although PHE, formerly known as EPS-like HE [41], should be considered in the differential diagnosis of EPS, it does not express CD34 and retains SMARCB1 expression. In addition, some PHEs are associated with a balanced translocation t(7;19)(q22;q13), resulting in a serpin family E member 1 (*SERPINE1*)–FosB proto-oncogene, AP-1 transcription factor subunit (*FOSB*) fusion [42,43]. FOSB expression is also useful for distinguishing PHE from EPS [44]. A subset of MPNSTs shows focal areas with an epithelioid morphology. In contrast to EPS, epithelioid MPNST is strongly and diffusely positive for S100 and SRY-box transcription factor 10 (SOX10). Synovial sarcoma can show large epithelioid and polygonal cells with abundant eosinophilic cytoplasm, particularly in its poorly differentiated form. However, synovial sarcoma does not express CD34. Additionally, synovial sarcoma is characterized by a t(X;18)(p11.2;q11.2) translocation, resulting in an SS18 subunit of the BAF chromatin remodeling complex (*SS18*)–SSX family member (*SSX*) fusion [45]. In our opinion, melanoma should be excluded by examining the expression patterns of melanocytic markers, such as SOX10 and human melanoma black 45 (HMB45), which are typically negative in EPS. EWS RNA Binding Protein 1 (*EWSR1*)-fusion-negative myoepithelial carcinoma can overlap morphologically (including rhabdoid features), immunophenotypically (epithelial marker expression with loss of SMARCB1), and genetically (*SMARCB1* biallelic deletions) [46]. However, myoepithelial carcinoma displays co-expression of S100 and epithelial markers. The corresponding histological and immunohistochemical characteristics are summarized in Table 1.

## 5. Pathogenesis

EPS is associated with complex karyotypes that lack specific chromosomal aberrations [47,48,49,50,51,52,53,54,55]. However, structural rearrangements of 22q11–12 and 8q, often as i(8)(q10), have been identified in several cases [48,50,51,53,54]. Subsequent spectral karyotyping analysis showed that 22q was the most frequently involved chromosome arm, with two cases displaying a similar t(10;22) translocation [56]. Most notably, loss of heterozygosity on chromosome 22q11 was demonstrated in 60% of cases in one study [57]. Comparative genomic hybridization analyses have also revealed recurrent gains of 11q13 and 22q [58,59]. There was no gene amplification or overexpression of cyclin D1 (*CCND1*), located on 11q13.3, although CCND1 protein expression was found in 96% of cases by immunohistochemistry [60].

Both classic and proximal-type EPSs harbor biallelic *SMARCB1* deletions or, less frequently, monoallelic *SMARCB1* deletions [16,46,61,62,63]. Additionally, heterozygous intragenic deletions or loss-of-function mutations of *SMARCB1* have been observed in only proximal-type EPS [64]. *SMARCB1*, also known as integrase interactor 1 (*INI1*), is located on 22q11.23 and encodes a core subunit of the SWI/SNF ATP-dependent chromatin remodeling complex, which is implicated in epigenetic regulation, cell cycle progression, and crosstalk between signaling pathways [65]. SMARCB1 is ubiquitously expressed in the nuclei of all normal human cells [66], with SWI/SNF complex mutations being present in 20% of human cancers [67]. Like EPS, ERRT also involves *SMARCB1* gene inactivation and subsequent loss of SMARCB1 nuclear protein expression [68]. Loss of expression of other SWI/SNF subunits, such as polybromo 1 (PBRM1), SWI/SNF-related BAF chromatin remodeling complex subunit ATPase 4 (SMARCA4), SWI/SNF-related BAF chromatin remodeling complex subunit C1 (SMARCC1), and SWI/SNF-related BAF chromatin remodeling complex subunit C2 (SMARCC2), has also been identified in EPS [69,70]. In one report, concurrent loss of SMARCB1 and PBRM1 expression was observed in 73.9% of cases [69]. In contrast, loss of SMARCA4, SMARCC1, and SMARCC2 expression was seen in a very small subset of SMARCB1-preserved proximal-type EPSs [70]. Most recently, Tokunaga et al. reported a case of a 50-year-old man with *SMARCA4*-deficient, *SMARCB1*-preserved EPS, who showed a notable response to nivolumab [71].

MicroRNAs (miRNAs) are small non-coding RNAs that act as endogenous regulators of gene expression by repressing translation or inducing messenger RNA (mRNA) degradation [72]. Several studies have suggested that oncogenic miRNAs, including miR-193a-5p, miR-206, miR-381, and miR-671-5p, might play essential roles in inhibiting *SMARCB1* mRNA expression in EPS [73,74,75]. Among these, the most effective miRNA was miR-206 [74]. Additionally, miR-765 was found to be significantly overexpressed in EPS, but further analysis indicated that it had no functional activity to reduce *SMARCB1* mRNA expression patterns [74]. Unfortunately, these results were not confirmed in other in vitro experiments [63]. Additional studies are required to elucidate the mechanism of loss of SMARCB1 expression in *SMARCB1*-preserved EPS cases.

The phosphoinositide-3 kinase (PI3K)/AKT/mechanistic (formerly mammalian) target of the rapamycin (mTOR) signaling pathway has also been shown to be activated in EPS [76,77]. Complete loss of phosphatase and tensin homolog (PTEN) expression was observed in 40% of cases in one report [76]. Additionally, expression of the phosphorylated form of 4E-binding protein 1 (4E-BP1) and S6 ribosomal protein at various levels was identified in all EPS tissue specimens analyzed [76]. Imura et al. reported that inhibiting the mTOR pathway with everolimus (an mTOR inhibitor) could suppress EPS cell growth but caused an increase in AKT expression via MET proto-oncogene, receptor tyrosine kinase (MET) action [77]. This result suggested the need to block multiple pathways with different agents. Indeed, combining agents to block both mTOR and MET signaling was more effective in inducing cell cycle arrest compared with using a single agent in the preclinical setting [77].

Epidermal growth factor receptor (EGFR) expression was detected in 93% of EPS cases regardless of subtype, although these cases showed no *EGFR* amplifications or kinase domain mutations [78]. Xie et al. also reported that EGFR expression was observed in 77% of cases, with increased EGFR expression levels similarly noted in two human EPS cell lines [76]. Moreover, EGFR activation was found to increase EPS cell proliferation and motility rates. Unfortunately, because AKT expression was sustained by the mTOR pathway, tumor arrest was not demonstrated following treatment with the EGFR inhibitor erlotinib alone [76]. Similarly, single-agent treatment with cetuximab, another EGFR inhibitor, failed to show significant activity in patients with advanced sarcoma [79]. These disappointing results suggested that EGFR blockade alone might be insufficient for effective tumor control. Indeed, combining agents to block both EGFR and mTOR signaling was significantly more effective compared with using a single agent in the preclinical setting [76].

Positive expression of MET and its ligand, hepatocyte growth factor, was found in nearly all the EPS tissue specimens and two human EPS cell lines examined [77,80]. Synovial sarcoma tissues are also known to express MET [80]. As mentioned above, MET inhibitors may be an effective therapeutic option for EPS.

Cyclin-dependent kinase inhibitor 2A (*CDKN2A*) deletions were observed in two EPS tissue specimens and two human EPS cell lines [63]. According to the Foundation Medicine database, *CDKN2A* alterations, including mainly copy number losses, were found in 20.8% of cases [81]. Loss of CDKN2A expression was also detected in 37% of cases [63]. It was suggested that loss of CDKN2A expression might occur independently of SMARCB1 deficiency in EPS.

Capping actin protein of muscle Z-line subunit beta (CAPZB) protein expression was detected in all eight EPS tissue specimens assessed using Western blot analysis [82]. In another study, immunohistochemistry experiments also demonstrated CAPZB protein expression in all 15 EPS tissue specimens examined and two human EPS cell lines [83]. Knockdown of CAPZB expression inhibited EPS cell growth, invasion, and migration. However, the relationship between SMARCB1 and CAPZB remains to be clarified in EPS pathogenesis.

Moderate-to-high expression levels of vascular endothelial growth factor (VEGF) have been described in EPS [84,85], suggesting a potential therapeutic target. Notably, VEGF-C expression was found in 96% of cases [85]. Increased VEGF-C levels are known to promote active intratumoral and peritumoral lymphangiogenesis and lymphatic tumor spread to regional lymph nodes [86,87]. However, Lahat et al. reported that increased VEGF-C expression patterns were insufficient to induce lymphatic metastasis in STSs, although EPS was not included in this study [88].

Dysadherin expression, which downregulates E-cadherin expression, was detected in 54% of cases, especially in proximal-type EPS [36]. Higher *dysadherin* mRNA expression levels were demonstrated in cell lines derived from proximal-type EPS compared with levels in those derived from classic EPS [36]. These results indicate that dysadherin expression is associated with poor prognosis in EPS. Moreover, a few studies have shown that E-cadherin expression was not observed in any EPS cases [36,85]. Therefore, we speculate that dysadherin may be a potential therapeutic target for treating EPS.

More recently, Shibui et al. reported that forkhead box protein M1 (FOXM1) expression was observed in all 38 EPS tissue specimens examined [89]. FOXM1 is a pro-proliferative transcription factor that is known to promote cell cycle progression at the G1/S and G2/M phases [90]. In addition, downregulation of FOXM1 was shown to reduce EPS cell proliferation, drug resistance to chemotherapeutic agents, migration, and invasion [89]. Moreover, cDNA microarray analysis demonstrated that FOXM1 could regulate cellular inhibitor of apoptosis protein 2 (cIAP2), an apoptosis inhibitor activated by the tumor necrosis factor alpha (TNF-alpha)-mediated NF-kappa-B signaling pathway [89]. These results suggest that FOXM1 inhibition may be a potential therapeutic option for EPS.

Recent DNA methylation and gene expression analyses have indicated molecular differences between classic and proximal-type EPSs [91]. Classic EPS showed positive expression of endothelial markers, such as SRY-box transcription factor 17 (SOX17), ERG, and N-cadherin. In contrast, proximal-type EPS exhibited MYC proto-oncogene, bHLH transcription factor (MYC) hyperactivation and GATA binding protein 3 (GATA3) expression, with extensive epigenetic rewiring associated with enhancer of zeste 2 polycomb repressive complex 2 subunit (*EZH2*) overexpression. Similarly, Rasmussen et al. reported biological differences between the two EPS subtypes, with distinguishable genes such as GLI family zinc finger 3 (*GLI3*), FYN proto-oncogene, Src family tyrosine kinase (*FYN*), and C-X-C motif chemokine ligand 12 (*CXCL12*) [92]. In that study, the authors also identified poly(A) binding protein cytoplasmic 1 (*PABPC1*) as a highly expressed target in EPS but did not observe an effect on autonomous cell viability in the interference experiment. These molecular details suggest that a histological subtype-tailored approach may be required in patients with EPS.

Frequent gene and protein alterations observed in EPS are summarized in Table 2.

## 6. Management

### 6.1. Localized Disease

Once STS is suspected, patients should be referred to a specialized sarcoma center. CT for detecting lung and lymph node metastases should be performed prior to definitive surgery. The standard approach for obtaining a histological diagnosis of a suspicious soft tissue mass is percutaneous core needle biopsy. Surgical resection is the standard treatment approach for nonmetastatic, localized EPS patients. A wide resection with negative margins (absence of microscopic residual tumor, R0) is the surgical procedure of choice. However, achieving R0 resection is more challenging for EPS involving the proximal region because of the anatomical constraints. Although preoperative treatment can allow limb-sparing surgery, amputation may be an option for EPS involving the distal region in selected cases.

As mentioned above, EPS has a high propensity for lymphatic metastasis. When lymph node metastasis is detected on preoperative imaging, most surgeons perform lymph node dissection during the initial surgery [93]. Given the risk of regional lymph node metastasis in some STS subtypes, there has been considerable interest in performing sentinel lymph node biopsy (SLNB) [93]. However, SLNB is not established as standard care for EPS. The SLNB positivity rates range from 0% to 14.2% in EPS [94]. Although suspicious nodes require pathological confirmation, the prognostic and therapeutic value of selective SLNB remains uncertain. We believe that any evaluation of SLNB in EPS should be done clearly using prospective clinical trials.

The goal of using radiation therapy (RT) in addition to surgical resection for localized STS is to improve local control. Callister et al. reported that seven (29%) of the 24 EPS patients receiving perioperative RT developed local recurrence [95]. In this study, the actuarial local control rate at 10 years was 63%. Wolf et al. reported that five (71%) of the seven primary EPS patients receiving adjuvant RT developed local recurrence [96]. Furthermore, adjuvant RT and chemotherapy had no impact on disease-free survival. Shimm et al. reported that one (17%) of the six EPS patients receiving perioperative RT had local recurrence [97]. In this study, the actuarial local control rate at seven years was 82%. Most recently, the antitumor efficacy of boron neutron capture therapy in EPS was demonstrated in an EPS-bearing animal model [98]. Further investigation is required to better define the optimal therapeutic approaches for localized EPS.

### 6.2. Advanced/Metastatic Disease

Strong evidence supporting systemic therapy for locally advanced or metastatic EPS is lacking. Most available data are from small retrospective studies, case reports, or single patients with EPS treated in all-comers STS clinical trials.

A recent multicenter, retrospective, real-world study involving 74 patients with advanced or metastatic EPS showed that the real-world objective response rate (rwORR) was 15% in the first-line treatment and 9% in the second-line treatment and beyond [99]. For patients receiving the first-line treatment, the median real-world progression-free survival (rwPFS) and median overall survival (OS) were 2.5 months and 15.2 months, respectively. For patients receiving the second-line treatment and beyond, the median rwPFS was 6.0 months. Unfortunately, significant adverse events were observed in approximately 51.4% of patients receiving chemotherapy, with febrile neutropenia (14%) and pain (10%) being the most common. The results of this study indicated only limited or temporary efficacy of the current chemotherapy.

Doxorubicin-based chemotherapy, either monotherapy or in combination with ifosfamide, is a standard first-line treatment for advanced/metastatic STS [100]. In the largest multicenter case series involving 115 patients with advanced/metastatic EPS, 85 patients (74%) were treated with doxorubicin-based regimens [101]. The ORR was 22% (19/85), with a median PFS of 6 months and median OS of 16 months. In the European Organization for Research on Treatment of Cancer (EORTC) clinical trial involving 27 EPS patients with metastatic disease, Touati et al. reported that the ORR was 12.5% (1/8) in patients treated with the doxorubicin and ifosfamide combination therapy and 0% (0/5) in patients treated with doxorubicin monotherapy [102]. A retrospective, multi-institutional analysis of 13 EPS patients showed that the median PFS was 3 months in patients treated with doxorubicin monotherapy and 8 months in patients treated with the doxorubicin and ifosfamide combination therapy, with a clinical benefit rate (CBR) of 46% [103]. Recently, for doxorubicin-based chemotherapy, Kashyap et al. reported a similar ORR (16%) in 12 patients with advanced EPS [104].

Gemcitabine-based chemotherapy, preferably in combination with docetaxel, is active and well tolerated in patients with advanced/metastatic STS [105]. In a retrospective, multi-institutional analysis of 12 EPS patients treated with the combination of gemcitabine and docetaxel, Pink et al. reported that the ORR and CBR were 58% (7/12) and 83% (10/12), respectively [103]. The median PFS was 8 months in all patients. The authors concluded that the gemcitabine and docetaxel combination therapy was effective for EPS, regardless of the line of therapy. Frezza et al. reported that the ORR of gemcitabine-based therapy was 27% (11/41) in patients with advanced/metastatic EPS, with a median PFS of 4 months and median OS of 19 months [101]. Recently, for the gemcitabine and docetaxel combination therapy, Kashyap et al. reported a lower ORR (20%) in five patients with advanced EPS [104].

Vinorelbine is a semisynthetic vinca alkaloid with a broad spectrum of antitumor activity [106]. Two case reports have described the benefit of vinorelbine in EPS patients [107,108]. One patient achieved a partial response (PR) with a duration of 27 months [107], while the other had a complete remission of pulmonary metastases with a durable response for four years [108]. Overall, these results indicate anecdotal clinical activity.

Recently, Xie et al. investigated the efficacy and tolerability of irinotecan-based chemotherapy in eight patients with advanced/metastatic EPS [109]. These patients received 54 treatment courses of the vincristine, irinotecan, and anlotinib (VIA) regimen. The ORR was 37.5% (3/8), with a median OS of 21.3 months. PFS could not be adequately assessed. The most common grade 3 or 4 adverse events included neutropenia, diarrhea, and nausea and vomiting. This combination therapy showed relatively high response rates but had a high incidence of adverse events.

Pazopanib is an oral multi-target tyrosine kinase inhibitor (TKI) with anti-angiogenic and anti-tumorigenic properties. In a multicenter case series, Frezza et al. demonstrated no objective responses in any of the 18 EPS patients treated with pazopanib, with a median PFS of 3 months and median OS of 14 months [101]. The results of this study were not satisfactory. Kashyap et al. reported that the ORR was 16% (1/6) in advanced EPS patients receiving pazopanib [104]. In contrast, there are sporadic reports of successful treatment with pazopanib in patients with advanced/metastatic EPS [110,111,112], with Touati et al. describing an ORR of 100% (2/2) with pazopanib as the first-line treatment and 11% (1/9) as the second- or later-line treatment [102]. Sunitinib, another multi-target TKI, showed long-term disease stabilization (>32 months) in a 34-year-old man with multiple metastatic EPS [113]. An open-label, single-arm phase 2 trial (SARC009) involving 109 advanced STS patients treated with dasatinib (a multi-target TKI) demonstrated that two (28%) of the seven EPS patients examined achieved an objective tumor response according to the Choi criteria, with a median PFS of 7.9 months [114]. However, the OS rate was only 21% at 2 years.

Trabectedin can be administered effectively and safely to patients with advanced STS in a second- or later-line setting [115]. A retrospective national analysis of 10 patients with advanced EPS treated with trabectedin as second-line treatment showed that the median PFS and median OS were 4.6 months and 12.0 months, respectively [116]. Moreover, the PFS rate was 70% at 3 months. In the EORTC clinical trial, Touati et al. reported that the ORR was 33.3% (1/3) in advanced/metastatic EPS patients receiving trabectedin [102]. Large prospective clinical trials are required to evaluate the role of trabectedin in the treatment of advanced/metastatic EPS.

As mentioned above, loss of SMARCB1 expression is seen in more than 90% of EPS cases. SMARCB1 loss leads to the unopposed, constitutive, oncogenic activation of EZH2. Tazemetostat is an oral selective EZH2 inhibitor. In an open-label, multicenter, phase 1 study (NCT01897571) involving three patients with advanced EPS treated with tazemetostat, stable disease (SD) was observed in two patients with SMARCB1-negative EPS with a prolonged duration of response (>20 months) [117]. A subsequent international, open-label, phase 2 basket study (NCT02601950) comprising 62 SMARCB1-negative EPS patients treated with 800 mg of tazemetostat twice daily demonstrated that the ORR, median PFS, and median OS were 15% (9/62), 5.5 months, and 19.0 months, respectively [118]. Most of the adverse events observed in the trial were grade 1 or 2. From the results of this NCT02601950 study, tazemetostat was approved by the United States Food and Drug Administration (FDA) for the treatment of adults and pediatric patients aged 16 years and older with metastatic or locally advanced EPS not eligible for complete resection. On March 9, 2026, however, Ipsen voluntarily withdrew tazemetostat from all markets directly managed by the company because a safety signal emerged in a phase 1b/3 SYMPHONY-1 trial for relapsed/refractory follicular lymphoma demonstrating secondary hematologic malignancies in 5.7% of treated patients. It should be clarified that this safety signal was not observed in the EPS cohort of the phase 2 study. A phase 1b/3 global, randomized, double-blind, placebo-controlled trial (NCT04204941) of tazemetostat in combination with doxorubicin in the first-line setting was stopped early. Recently, Zhou et al. reported a phase 2 trial of SHR2554, another oral selective EZH2 inhibitor, in 14 patients with advanced EPS [119]. Among these patients, three (21.4%) achieved a PR, with SD observed in eight (57.1%). Adverse events in the trial were generally mild. Most recently, Fan et al. reported an open-label, multicenter, phase 1 trial (NCT04390737) of HH2853, an oral selective EZH1/2 dual inhibitor, in 32 patients with advanced EPS [120]. The ORR was 31.3% (10/32), with a median PFS of 16.0 months. However, the median OS was not reached. The safety profile was acceptable, with a recommended phase 2 dose of 400 mg twice daily. Further investigations should focus on larger-scale randomized controlled trials to validate these results.

Immunotherapy is an emerging treatment approach for a subset of SMARCB1-deficient STSs [121]. There is growing interest in exploring the potential of applying immune checkpoint inhibitors (ICIs) for the treatment of advanced/metastatic EPS. The FDA has approved three different categories of ICIs: programmed cell death 1 (PD-1) inhibitors (nivolumab, pembrolizumab, and cemiplimab), programmed cell death ligand 1 (PD-L1) inhibitors (atezolizumab, durvalumab, and avelumab), and cytotoxic T-lymphocyte-associated protein 4 (CTLA-4) inhibitor (ipilimumab). EPS tends to express high levels of PD-L1 [122,123]. Moreover, Haefliger et al. reported a predominance of CD8+ lymphocytes and M2 macrophages in the EPS tumor microenvironment [124]. Most recently, Ngo et al. also reported that classic EPS was enriched in cytotoxic and CD8+ T cells, natural killer cells, and M1 macrophages, whereas proximal-type EPS was enriched in M2 macrophages [125]. These findings suggest that EPS may be considered to be an immunogenic neoplasm that can benefit from ICIs. An open-label, single-arm, phase 1/2 trial (KEYNOTE-051) showed that one patient with PD-L1-positive EPS achieved a PR with pembrolizumab [126]. In a non-randomized, open-label, phase 2, basket trial (NCT03012620) involving six patients with EPS treated with pembrolizumab, one (17%) achieved a complete response, and three (50%) had prolonged SD [127]. Wang et al. also reported the efficacy of pembrolizumab in a 28-year-old man with advanced EPS displaying high PD-L1 expression levels (>50%) [128]. The patient achieved a PR after six cycles of pembrolizumab. There are a few clinical studies regarding the use of nivolumab in advanced/metastatic EPS patients [71,129]. In a retrospective series, a 24-year-old man with metastatic EPS achieved a PR after four cycles of nivolumab; however, the response was not durable because the patient progressed after the next four cycles, with another patient with EPS displaying progressive disease at the first evaluation of nivolumab [129]. An interesting EPS case of a remarkable response to nivolumab was recently reported [71]. This case showed both SMARCA4 deficiency and a high tumor mutation burden. In the open-label, non-comparative, randomized, phase 2 trial (Alliance A091401), the combination of nivolumab and ipilimumab appeared to be superior to nivolumab monotherapy in other metastatic sarcomas [130]. Notably, Pecora et al. reported that a 17-year-old man with stage IV refractory end-stage EPS had a complete and durable response to ipilimumab and nivolumab combination therapy [131]. The combination of ipilimumab and nivolumab is under further evaluation in SMARCB1-negative pediatric cancers, including EPS (NCT04416568). Recently, camrelizumab, a PD-1 inhibitor, was used in a 30-year-old man with metastatic EPS showing over 30% of PD-L1 positivity and a high number of tumor-infiltrating lymphocytes [132]. The patient had a complete and durable response after 18 cycles of camrelizumab. Beyond ICIs, autologous immune enhancement therapy (AIET) may also be beneficial to EPS patients. Ratnavelu et al. reported that a 35-year-old man with advanced EPS received seven AIET infusions using expanded natural killer cells and T cells, achieving SD without any side effects [133]. However, it should be kept in mind that the evidence for ICIs and autologous immunotherapy remains investigational or anecdotal, with no systematic benefit shown thus far.

Combination therapies may ultimately prove to be more efficacious. A combination of ICIs and multi-target TKIs for the treatment of advanced EPS has been studied in clinical trials [134,135,136]. A single-center, single-arm, phase 2 trial showed that one patient with advanced EPS achieved a prolonged PR with a combination of axitinib and pembrolizumab [134]. A multicenter, single-arm, phase 1b/2 trial showed no objective responses in seven patients with advanced EPS receiving a combination of nivolumab and sunitinib [135]. A single-center, phase 1b trial revealed that one patient with advanced EPS had SD with a combination of dasatinib and ipilimumab [136]. Representative clinical trials for advanced/metastatic EPS are summarized in Table 3.

## 7. In Vivo Models

In vivo models for EPS are available for preclinical research and therapeutic screening, primarily categorized into cell line-derived xenograft (CDX), patient-derived xenograft (PDX), organoid-derived xenograft (ODX), and genetically engineered mouse models (GEMMs). Currently, there are more than 15 CDX models [137], including FU-EPS-1 [55]. Compared to CDX, PDX can offer a more advantageous platform for preclinical drug screening and biomarker identification. Recently, a few PDX models have been established from patients with classic and proximal EPSs [138,139,140]. In 2022, Wakamatsu et al. first reported that two ODXs were successfully established from patients with classic EPS [141]. Unfortunately, well-established GEMMs for EPS appear to still be lacking.

## 8. Study Limitations

This review has some limitations that should be acknowledged. The rarity of EPS has limited the number of published case series and the size of available cohorts. These conditions impeded the design of complex studies. A substantial portion of the data reviewed came from small case series or individual case reports, reducing the level of evidence in our casuistry, particularly in the therapeutic section. Moreover, retrospective designs may introduce selection and information bias. Finally, exclusion of non-English publications and conference abstracts without full-text articles may have omitted some preliminary or regional data.

Taken together, these limitations highlight the need for more standardized, longitudinal, and multicenter research involving larger, more diverse samples.

## 9. Conclusions and Future Directions

EPS is an ultra-rare malignant mesenchymal neoplasm posing significant diagnostic and therapeutic challenges. Proximal-type EPS behaves more aggressively than classic EPS. Compared with other STSs, EPS has a higher rate of regional lymph node metastasis. Loss of SMARCB1 is an important diagnostic feature, but it is neither entirely specific nor an established predictive biomarker. Wide surgical resection is the mainstay of treatment for localized EPS, although perioperative RT may be considered in appropriately selected patients. Conventional chemotherapy has shown modest, generally short-lived activity in retrospective cohorts. The withdrawal of tazemetostat has created a significant gap in treatment options. Immunotherapy is still under evaluation and lacks a validated biomarker. Ongoing collaboration between sarcoma centers, international prospective registries, central pathology review, histology-specific adaptive trials, molecular stratification, and long-term safety monitoring of epigenetic therapies will be critical to achieving satisfactory clinical outcomes in locally advanced or metastatic EPS cases.

Lastly, the cellular origin of EPS remains undefined. It also remains unknown whether classic and proximal-type EPSs arise from the same or different cellular populations. Further research is crucial to advancing our understanding of EPS biology.

## Figures and Tables

**Table 1:** Histological and immunohistochemical characteristics of epithelioid sarcoma and its major differential diagnoses.

Entity	Histological Features	Immunohistochemical Features
EPS	Epithelioid cells, plump spindle-shaped cells (classic type) and large polygonal cells (proximal type) with abundant eosinophilic cytoplasm, rhabdoid cells (especially proximal type)	Cytokeratins (+), EMA (+), CD34 (+, 52–73%), ERG (+, 38–68%), FLI1 (+, 93%), CA125 (+, 91%), S100 (−), loss of SMARCB1 (over 90%)
NG	Epithelioid histiocytes, lymphocytes, and occasionally multinucleated giant cells	Cytokeratins (−), EMA (−)
UC	Large epithelioid and round cells	Cytokeratins (+), EMA (+), CD34 (−)
ERRT	Rhabdoid cells with globoid and hyaline or eosinophilic intracytoplasmic inclusions	Cytokeratins (+), EMA (+), ERG (-), CD99 (+), synaptophysin (+), GPC3 (+), SALL4 (+), loss of SMARCB1
EA	Large, mildly to moderately pleomorphic epithelioid cells with abundant eosinophilic cytoplasm	Cytokeratins (variable), EMA (variable), CD34 (variable), ERG (+), CD31 (+), retention of SMARCB1
PHE	Plump spindle cells with abundant eosinophilic cytoplasm and sometimes epithelioid cells	Cytokeratins (+), EMA (typically negative), CD34 (-), ERG (+), FLI1 (+), FOSB (+), SMA (focal), retention of SMARCB1
EMPNST	Plump, epithelioid cells with abundant eosinophilic cytoplasm	S100 (+), SOX10 (+), loss of SMARCB1 (50%)
SS	Epithelioid and polygonal cells with abundant eosinophilic cytoplasm	Cytokeratins (+), EMA (+), CD34 (−), TLE1 (+), reduction of SMARCB1 (69%)
Melanoma	Epithelioid and spindle cells with marked cytologic atypia, intracytoplasmic pigment	SOX10 (+), HMB45 (+), S100 (+), typically negative for epithelial markers
MC	Myoepithelial cells consisting of epithelioid, spindled, plasmacytoid, and clear cells	Cytokeratins (+), EMA (+), S100 (+), SOX10(+), SMA (+), loss of SMARCB1 (10–40%)

EPS: epithelioid sarcoma; NG: necrobiotic granuloma; UC: undifferentiated carcinoma; ERRT: extrarenal rhabdoid tumor; EA: epithelioid angiosarcoma; PHE: pseudomyogenic hemangioendothelioma; EMPNST: epithelioid malignant peripheral nerve sheath tumor; SS: synovial sarcoma; MC: myoepithelial carcinoma; EMA: epithelial membrane antigen; ERG: ETS transcription factor ERG; FLI1: Fli-1 proto-oncogene, ETS transcription factor; SMARCB1: switch/sucrose nonfermentable (SWI/SNF)-related BAF chromatin remodeling complex subunit B1; GPC3: glypican 3; SALL4: spalt-like transcription factor 4; FOSB: FosB proto-oncogene, AP-1 transcription factor subunit; SMA: smooth muscle actin; SOX10: SRY-box transcription factor 10; TLE1: transducin-like enhancer of split 1; HMB45: human melanoma black 45.

**Table 2:** Frequent gene and protein alterations in epithelioid sarcoma.

Gene/Protein	Alterations	References
*SMARCB1*	Biallelic or, less frequently, monoallelic deletions	[16,46,61,62,63]
PTEN	Complete loss of expression	[76]
EGFR	Expression but no gene amplifications or mutations	[78]
MET	Expression	[80]
*CDKN2A*	Mainly copy number losses	[81]
CAPZB	Expression	[82,83]
VEGF-C	Expression	[85]
Dysadherin	Expression	[36]
FOXM1	Expression	[89]
*PABPC1*	Overexpression	[92]

SMARCB1: switch/sucrose nonfermentable (SWI/SNF)-related BAF chromatin remodeling complex subunit B1; PTEN: phosphatase and tensin homolog; EGFR: epidermal growth factor receptor; MET: MET proto-oncogene, receptor tyrosine kinase; CDKN2A: cyclin-dependent kinase inhibitor 2A; CAPZB: capping actin protein of muscle Z-line subunit beta; VEGF-C: vascular endothelial growth factor C; FOXM1: forkhead box protein M1; PABPC1: poly(A) binding protein cytoplasmic 1.

**Table 3:** Representative clinical trials including epithelioid sarcoma patients.

NCT Identifier	Drugs	Phase	Population	Status	Status Update Date
NCT04390737	HH2853 (EZH1/2 inhibitor)	1/2	EPS, FL, PTCL, advanced solid tumor	Recruiting	January 2026
NCT04416568	Nivolumab (anti-PD-1) + Ipilimumab (anti-CTLA-4)	2	EPS, MRT, RTK, ATRT, chordoma (poorly differentiated or dedifferentiated), other INI1- or SMARCB1-negative malignant tumors	Active, not recruiting	June 2026
NCT05407441	Tazemetostat * (EZH2 inhibitor) + Nivolumab + Ipilimumab	1/2	EPS, MRT, RTK, ATRT, chordoma, INI1 (SMARCB1)-deficient primary CNS malignant tumors, SMARCA4-deficient primary CNS malignant tumors	Active, not recruiting	April 2026
NCT05286801	Tiragolumab (anti-TIGIT) + Atezolizumab (anti-PD-L1)	1/2	EPS, ATRT, chordoma (poorly differentiated), kidney medullary carcinoma, malignant solid neoplasm	Active, not recruiting	July 2026
NCT03069378	T-VEC (oncolytic viral therapy) + Pembrolizumab (anti-PD-1)	2	EPS, sarcoma, cutaneous angiosarcoma	Active, not recruiting	June 2026
NCT04705818	Durvalumab (anti-PD-L1) + Tazemetostat *	2	Advanced STS including EPS, advanced solid tumor, advanced colorectal carcinoma, advanced pancreatic adenocarcinoma	Completed	March 2026
NCT04095208	Nivolumab + Relatlimab (anti-LAG-3)	2	Adult STS including EPS, advanced cancer	Completed	April 2026
NCT04204941	Tazemetostat * + Doxorubicin	1b/3	Advanced EPS, advanced STS	Terminated	January 2026

NCT Identifier: ClinicalTrials.gov identifier; EZH1/2: enhancer of zeste 1/2 polycomb repressive complex 2 subunit; PD-1: programmed cell death 1; CTLA-4: cytotoxic T-lymphocyte-associated protein 4; TIGIT: T-cell immunoreceptor with Ig and ITIM domains; PD-L1: programmed cell death ligand 1; T-VEC: talimogene laherparepvec; LAG-3: lymphocyte-activation gene 3; EPS: epithelioid sarcoma; FL: follicular lymphoma; PTCL; peripheral T cell lymphoma; MRT: malignant rhabdoid tumor; RTK: rhabdoid tumor of the kidney; ATRT: atypical teratoid/rhabdoid tumor; INI: integrase interactor 1; SMARCB1: switch/sucrose nonfermentable (SWI/SNF)-related BAF chromatin remodeling complex subunit B1; CNS: central nervous system; SMARCA4: SWI/SNF-related BAF chromatin remodeling complex subunit ATPase 4; STS: soft tissue sarcoma. * Tazemetostat is no longer routinely available.

## Data Availability

No new data were created or analyzed in this study. Data sharing is not applicable to this article.
